# Fostering Academic Inclusion and Representation: Enhancing Research Capacity for Black Nursing Academics in UK Universities—A Qualitative Multi‐Study Protocol

**DOI:** 10.1111/jan.70354

**Published:** 2025-11-05

**Authors:** Yetunde Ataiyero, Shenile Lindo, Odinaka Ani, Chinenye Ubah, Chinenye Anetekhai, Odunayo Kolawole Omolade, Love Onuorah, Alisen Dube, Sarahjane Jones

**Affiliations:** ^1^ Centre for Health Innovation, School of Health, Education, Policing and Sciences, University of Staffordshire Stoke‐on‐Trent UK; ^2^ Department of Nursing, School of Health Sport and Bioscience, University of East London London UK; ^3^ Faculty of Health, Medicine and Social Care, Anglia Ruskin University Cambridge UK; ^4^ School of Nursing and Midwifery, Birmingham City University Birmingham UK

**Keywords:** Black nursing academics, diversity, equity, higher education, inclusion, nursing, nursing education, nursing research, protocol, research capacity

## Abstract

**Background:**

Nursing as a profession remains underrepresented in research leadership, funding success and scholarly authorship globally, which limits its influence on policy and practice. Within this broader context, racially minoritised nursing academics, including Black academics, face additional inequities that further hinder their visibility and progression. Evidence from the United States, Canada and Australia highlights persistent barriers to research careers and leadership opportunities for Black nurses. In the United Kingdom, these disparities are particularly evident: Black nursing academics face barriers to conducting research while in the wider National Health Service workforce, Black nurses are twice less likely than their White counterparts to be promoted. Together, these patterns constrain career progression and hinder the development of culturally competent healthcare education and practice.

**Aim:**

To explore the barriers to conducting research among Black nursing academics working in UK universities that are not traditionally research intensive, and to co‐create pragmatic, theory‐informed recommendations for enabling supportive and equitable research environments.

**Design:**

A qualitative multi‐study design underpinned by Intersectionality Theory and The Silences Framework.

**Methods:**

Two work packages are proposed. Work Package 1 will use semi‐structured interviews to explore the experiences and barriers of conducting research among up to 15 Black nursing academics based at UK universities that are not research‐intensive. Work Package 2 will adopt a modified Delphi methodology, engaging key collaborators in two rounds of online codesign workshops. Findings from Work Package 1 will inform structured discussions in which collaborators will develop theory‐informed, pragmatic recommendations to strengthen research capacity and engagement among Black nursing academics.

**Conclusion:**

This study will address the persistent underrepresentation of Black nursing academics in research. While grounded in the UK, the anticipated outputs will have wider applicability, informing policy, shaping institutional strategies and guiding future research priorities across diverse academic and healthcare systems worldwide.

## 1 Introduction

Black academics in the United Kingdom (UK) continue to face systemic barriers that hinder their full participation in research, restrict access to funding opportunities and impede career advancement. Despite national commitments to equity and inclusion, persistent disparities in representation, research leadership and funding success rates persist (National Institute for Health and Care Research 2025; UK Research and Innovation 2025). These inequities are particularly pronounced for Black nursing academics, who experience a form of dual marginalisation: first, within the wider academic landscape and second, within the nursing profession, which itself remains underrepresented in research and scholarly leadership (Iheduru‐Anderson 2025). This persistent underrepresentation creates a glass ceiling effect on the career progression of Black scholars, contributing to an epistemic imbalance that limits the nursing profession's capacity to deliver high‐quality, culturally responsive care to the increasingly diverse populations.

Although situated in the UK, the challenges addressed in this study resonate internationally. In the United States, Black academics remain significantly underrepresented in research leadership roles and face structural barriers to progression (Griffin 2020). In Canada and Australia, scholars have documented systemic racism and inequities affecting racially minoritised and Indigenous academics across disciplines, including health and nursing (Henry et al. 2017; Durey and Thompson 2012). Global reviews of nursing highlight persistent gaps in research capacity and leadership, limiting nurses' ability to shape policy and practice (World Health Organization 2020). Taken together, these findings indicate that inequities in nursing academia are both nationally specific and part of a wider international pattern.

## 2 Background

Data from the Higher Education Statistics Agency (HESA) indicate that in the 2023/24 academic year, only 250 of the 25,670 professors in the UK were Black, representing < 1% of all professors (Higher Education Statistics Agency 2025). This stark underrepresentation in senior academic roles mirrors international trends: in the United States, only about 7% of full‐time academics are Black, compared with 72% who are White, 13% Asian and 6% Hispanic (National Center for Education Statistics 2024). Evidence further shows that Black academics are less likely to hold leadership positions, secure permanent contracts or obtain research funding (Bhopal 2019; Arday 2018). Within the UK National Health Service (NHS), similar inequities persist, with Black nurses twice as likely to be overlooked for promotion compared to White colleagues (Mitchell 2022). At a global level, nurses remain significantly underrepresented in research and scholarly authorship, limiting the profession's ability to shape health policy and evidence‐based practice (Royal College of Nursing 2024). The World Health Organization highlights persistent underinvestment in nursing leadership and research capacity, which constrains the profession's influence on health systems and policy development (World Health Organization 2020).

International evidence underscores that these challenges are systemic. In the United States, Black nurses remain markedly underrepresented in senior academic roles, with only about 9% of nursing academics identifying as Black compared to 13% of the national population (National League for Nursing 2016; Iheduru‐Anderson 2020). Institutional practices such as exclusion from leadership pathways and limited access to mentorship further constrain progression (DeWitty and Murray 2020). Similar patterns are evident in Canada, where systemic racism and discriminatory practices have been documented, with Black nurses reporting exclusion from leadership opportunities and a scarcity of Black professors (Jefferies et al. 2022; Registered Nurses' Association of Ontario Black Nurses Task Force 2022). In Australia, research highlights the compounded disadvantage faced by Black African migrant nurses and the persistent underrepresentation of Aboriginal and Torres Strait Islander scholars in academic leadership, despite national cultural safety frameworks (Dywili et al. 2021). Collectively, these findings reveal entrenched inequities that limit visibility, influence and leadership opportunities for racially minoritised nursing academics globally.

Employment structures amplify these disparities. Black academics are disproportionately employed in teaching‐focused roles, limiting opportunities to build research portfolios and secure competitive grants (University and College Union 2021; Franssen et al. 2024). Employment insecurity compounds these challenges. Forty per cent of Black academics are employed on fixed‐term contracts, compared with 32% across the UK higher education sector (University and College Union 2021), restricting their capacity for long‐term planning and reducing access to institutional research support. Comparable trends have been reported in Australia and Canada, where Indigenous and racially minoritised academics are concentrated in precarious academic roles with limited opportunities for sustained research careers (Henry et al. 2017; Durey and Thompson 2012).

Funding disparities further entrench these inequities. In 2020–2021, only 13% of Black applicants were successful in UK Research and Innovation (UKRI) funding applications, compared with 29% of White applicants, with Black academics representing just 1% of successful Principal Investigators (UK Research and Innovation 2020). These gaps are mirrored in the Research Excellence Framework 2021 (REF2021), the UK's national system for assessing research quality and allocating public research funding. REF2021 results showed that only 53% of Black academics were submitted, compared with 75% of White and 80% of Asian academics (Research Excellence Framework 2023). Within Main Panel A, which covers medicine, health services, nursing and biological sciences, the disparities were even more pronounced, with submission rates of 72.3% for White academics, 79.3% for Asian academics and only 46.5% for Black staff (Research Excellence Framework 2023). These patterns are not unique to the UK; they reflect broader international trends where research assessment and funding systems reproduce inequities for racially minoritised academics (Nguyen et al. 2023; Petersen 2021).

In UK nursing academia, the Royal College of Nursing reports 311 professors across 79 UK universities but does not disaggregate these data by ethnicity (Royal College of Nursing 2024). This lack of transparency obscures the true extent of underrepresentation. Similar challenges have been observed internationally, with nursing professorships often dominated by White academics, limiting visibility and leadership opportunities for Black and other racially minoritised scholars (Iheduru‐Anderson 2020). To address this evidence gap in the UK, the lead author of this article is undertaking an independent study using Freedom of Information requests to estimate the proportion of Black nursing academics with significant research responsibility, with the aim of informing more effective equity‐focused strategies.

The UK's research landscape further shapes these dynamics. The Russell Group comprises 24 self‐selected public research universities renowned for world‐class research, high‐quality teaching and strong connections with industry and the public sector. These universities receive a significant share of national research funding and enjoy high prestige both nationally and internationally. The Russell Group is broadly comparable to the Ivy League in the United States, the U15 Group of Canadian Research Universities or the Group of Eight in Australia. Evidence suggests that academics based at such research‐intensive institutions are more likely to secure research funding (Schneider et al. 2024). It is therefore reasonable to infer that the majority of Black academics who submitted to REF2021 were affiliated with Russell Group universities. However, there is a notable gap in subject representation: of the 82 UK universities offering nursing and midwifery programmes, only 14 belong to the Russell Group (Complete University Guide 2024). This imbalance underscores potential disparities in access to research opportunities and visibility for these disciplines within the UK's most research‐intensive environments. Comparable patterns are evident internationally, where health and nursing disciplines are often underrepresented in the most research‐intensive institutions, reinforcing inequities in access to funding and recognition.

The purpose of this study is to explore the specific challenges faced by Black nursing academics working in UK universities that are not members of the Russell group, the national association of research‐intensive universities. By codesigning practical, evidence‐informed recommendations, the study seeks to strengthen research capacity and visibility among this underrepresented group. By critically examining systemic barriers, including unequal access to research opportunities, disparities in funding and limited pathways for career progression, the study aims to contribute to a more inclusive academic environment. Its findings are expected to inform strategies relevant across diverse academic and healthcare systems worldwide.

## 3 Study Aim and Objectives

### 3.1 Aim

This study aims to explore the barriers to conducting research among Black nursing academics working at non‐Russell group UK universities, and to co‐create pragmatic, theory‐informed recommendations for enabling supportive and equitable research environments.

### 3.2 Objectives

The specific objectives are to:

1. Identify and understand the key barriers and challenges faced by Black nursing academics in conducting research.
2. Explore effective strategies and practices that can be implemented to address these barriers and create supportive research environments.
3. Co‐create theory‐informed, pragmatic recommendations to foster enabling and equitable research environments for Black nursing academics in non‐Russell Group UK universities, with consideration of their transferability to other Black academics and racialised researchers in similar institutional contexts.

## 4 Methods/Methodology

### 4.1 Study Design

This study adopts a qualitative multi‐study design, structured into two interconnected work packages. Work Package 1 will use semi‐structured qualitative interviews to explore the experiences of Black nursing academics in depth. Work Package 2 will apply a modified Delphi methodology within codesign workshops to collaboratively develop and refine practical, evidence‐informed recommendations. This combination of methods enables rich exploration of lived experiences, followed by an iterative process of consensus building to produce actionable strategies.

### 4.2 Study Setting

Nursing education in the UK is predominantly delivered outside the Russell Group of research‐intensive universities. To address the underrepresentation of Black academics in research from these institutions, participants for Work Package 1 will be recruited primarily from Non‐Russell Group universities. This focus will ensure a broad spectrum of experiences is captured.

In Work Package 2, key collaborators for the codesign workshops will be drawn from both Russell Group and non‐Russell Group universities. This inclusive approach will provide a diversity of perspectives, enabling the co‐creation of recommendations that are relevant and applicable across different academic contexts.

In both work packages, efforts will be made to draw participants from all four nations of the UK, ensuring that the study reflects a breadth of regional contexts and experiences.

### 4.3 Theoretical Framework

This study is underpinned by the Intersectionality Theory (Crenshaw 2017, 1989, 1991) and The Silences Framework (Serrant‐Green 2011; Serrant 2020).

The Silences Framework values personal experiences and amplifies the voices of marginalised groups, creating space for discourses that are often excluded from mainstream research. It comprises five cyclical stages: *Working in Silences* (contextualisation), *Hearing Silences* (location), *Voicing Silences* (verbalisation), *Working with Silences* (discussion) and *Planning for Silences* (recommendations). These stages collectively provide a structure for uncovering and amplifying seldom‐heard perspectives throughout both research processes and outputs.

Intersectionality Theory examines how multiple, overlapping identities, such as race, gender and professional status interact to produce unique experiences of social inequality. Crenshaw (1989, 1991) identifies three key dimensions: *structural intersectionality*, which highlights how systems of power such as racism and sexism compound disadvantage; *political intersectionality*, which reveals how policies or institutional responses can marginalise individuals whose identities cross categories; and *representational intersectionality*, which addresses cultural narratives and stereotypes that reinforce oppression. These dimensions provide a lens for analysing the compounded inequities that Black nursing academics experience within higher education and healthcare research.

These frameworks are applied at multiple points in the study. They inform the design of the interview guide, with the Silences Framework guiding the inclusion of prompts that encourage participants to share experiences that might otherwise remain hidden, and Intersectionality Theory shaping questions that probe how identity categories interact in academic and professional contexts.

The two frameworks converge during a theory integration stage that precedes Framework Analysis (Hackett and Strickland 2019). At this point, the Silences Framework ensures that marginalised narratives are systematically incorporated into the analytical matrix, while Intersectionality Theory provides the interpretive lens to examine how structural inequalities manifest at the intersections of race, gender and professional identity. This integration strengthens the explanatory power of Framework Analysis in Work Package 1 and guides collaborative synthesis in Work Package 2.

**FIGURE 1:**
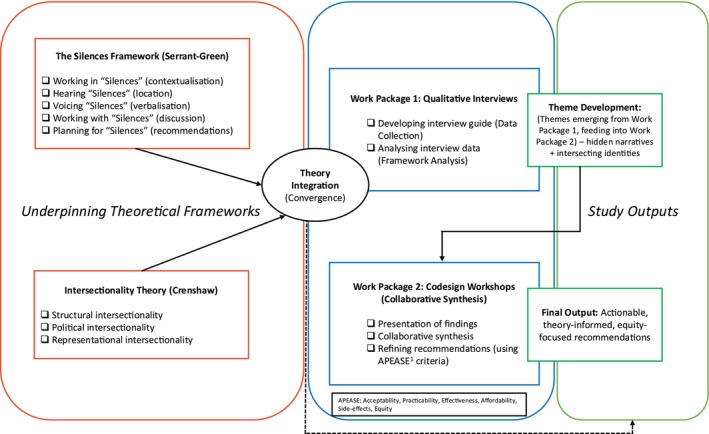
Integration of the Silences Framework and Intersectionality Theory across the study. The Silences Framework (Serrant‐Green 2011; Serrant 2020) comprises five stages (Working in, Hearing, Voicing, Working with and Planning for Silences), while Intersectionality Theory (Crenshaw 1989, 1991, 2017) encompasses structural, political and representational intersectionality. These frameworks converge in a theory integration stage that informs both Work Package 1 (interview guide development and framework analysis) and Work Package 2 (codesign workshops). Themes emerging from Work Package 1 feed into Work Package 2, where recommendations are collaboratively refined using the APEASE criteria. The final outputs: Actionable, theory‐informed, equity‐focussed recommendations also feed back into the theoretical foundations, reflecting the iterative and reflexive nature of the study. APEASE: Acceptability, Practicability, Effectiveness, Affordability, Side‐effects/Safety, Equity (Atkins 2016).

Bringing these frameworks together ensures that Black nursing academics remain central to the study, integrated as knowledge producers rather than positioned as mere participants. This approach seeks to avoid identity fragmentation, acknowledges the diverse challenges faced and builds a robust evidential base to challenge entrenched norms and strengthen calls for change. The integration and application of these frameworks across the study design are illustrated in Figure 1.

### 4.4 Work Package 1: Qualitative Interviews

This work package will use a semi‐structured interviewing approach to explore the barriers faced by Black nursing academics conducting research in non‐Russell Group UK universities.

#### 4.4.1 Objectives

To identify and understand the key barriers and challenges faced by Black nursing academics in non‐Russell group universities in conducting research.

#### 4.4.2 Sample Size

Up to 15 one‐to‐one, in‐depth, semi‐structured interviews will be conducted.

#### 4.4.3 Eligibility Criteria

The term *Black*, as used in this study, refers to individuals of Black African, Black Caribbean or Black mixed heritage who self‐identify as such.

Participants will be eligible for inclusion if they:

1. Are of Black African, Black Caribbean or Black mixed heritage and identify as such,
2. Are registered with the UK Nursing and Midwifery Council (NMC),
3. Are currently employed on an academic contract at a UK higher education institution,
4. Are based at a Non‐Russell Group university in the UK,
5. Were educated either in the UK or internationally,
6. May or may not hold significant responsibility for research, as defined in REF guidance: staff for whom explicit time and resources are made available to engage actively in independent research, and for whom this is an expectation of their role (Dayson 2019).

Recruiting individuals who meet these criteria is essential for exploring how Black nursing academics navigate the research landscape within non‐Russell Group UK universities. Their inclusion will allow for a deeper understanding of the systemic inequalities and institutional barriers that persist within the research and innovation ecosystem. In doing so, this study seeks to contribute to ongoing efforts to eliminate structural inequities and promote a more inclusive, equitable academic environment.

#### 4.4.4 Participant Sampling and Recruitment

An adaptive recruitment strategy will be employed, drawing on purposive, snowballing and stratified sampling techniques to achieve maximum variation in the study sample.

Purposive sampling will guide the initial selection of participants who can provide rich, diverse insights into the research topic (Palinkas et al. 2015). The study will target Black nursing academics employed at non‐Russell Group universities across the UK, ensuring variation across career stages, genders and educational backgrounds.

Snowballing sampling will complement this by encouraging early participants to recommend colleagues who meet the eligibility criteria but differ in key characteristics such as geographical location, research engagement and career stage. Snowballing is a recognised method for reaching individuals who may be marginalised or less visible within institutional networks (Kirchherr and Charles 2018). This approach will help mitigate selection bias and ensure the participant pool is not limited to familiar or accessible networks.

Given the disproportionate concentration of Black nursing academics in England, a stratified recruitment approach will be used to enhance representation from Scotland, Wales and Northern Ireland. Recruitment efforts will be continuously monitored and adjusted to address any imbalances, preventing overrepresentation from specific institutions or regions. Recruitment strategies will be refined iteratively throughout the study to address any underrepresentation and ensure a heterogeneous sample that reflects a wide range of experiences.

To maximise engagement and visibility, the research team will collaborate with key organisations, such as the Council of Deans of Health, the Nursing and Midwifery Council and the Society of Black Academics. Recruitment will also be supported by targeted dissemination via professional social media platforms to extend reach across the UK academic community. A study poster summarising key information will be used to support recruitment. It will include a QR code linking to an expression of interest form and the online participant information sheet, as well as the Principal Investigator's contact details for queries.

On behalf of the University of Staffordshire, email invitations will be sent to potential participants who meet the eligibility criteria, inviting them to take part in a one‐to‐one, in‐depth, semi‐structured, online interview. These interviews will explore individual experiences and barriers to research engagement among Black nursing academics. To signify interest, potential participants will be required to complete an expression of interest form, providing contact details with which the research team could reach them. The email will also include a participant information sheet outlining the purpose of the study and participants' rights to participation and withdrawal. The research team will be available to clarify any questions to support informed decision‐making.

Participation will be voluntary, and potential participants will be given a minimum of 24 h to consider their willingness to participate in the interviews before completing an electronic consent form for research records. Verbal consent will also be obtained and recorded on the day of the interview. In recognition of their time and contribution, participants will receive a £25 voucher.

#### 4.4.5 Data Collection

An interview schedule, informed by the study aim, relevant literature and the underpinning theoretical frameworks, will guide all interviews. The Principal Investigator, who is of Black ethnicity and a registered nurse, will conduct the interviews in a culturally sensitive and appropriate manner, recognising and respecting the cultural norms, values and lived experiences of participants.

In the unlikely event that the Principal Investigator is unavailable, Co‐Investigator 1, who is also of Black ethnicity and a registered nurse will conduct the interviews to ensure continuity in cultural competence. Should both the Principal Investigator and Co‐Investigator 1 be unavailable, other members of the wider project management team who are Black nursing academics are well‐positioned to undertake this role.

Only if none of the aforementioned individuals are available will Co‐Investigator 2, who is of White British ethnicity, conduct the interviews. To support cultural sensitivity in this instance, the interview schedule has been designed as a detailed script, enabling respectful, inclusive and contextually appropriate discussions regardless of who facilitates them.

To enhance accessibility and inclusivity, interviews will be conducted online via Microsoft Teams at a mutually convenient time, accommodating participants with caring responsibilities or other commitments. Each interview is expected to last up to 60 min.

The interview schedule includes structured wording, prompts and guidance on tone and approach, supporting consistency across interviews while preserving the depth, richness and flexibility of qualitative inquiry. It also provides guidance on establishing rapport, navigating sensitive topics and responding appropriately to any signs of participant distress or discomfort. Interviewers will draw on their professional and lived experience as registered nurses to create a safe, affirming environment for participants throughout the process.

#### 4.4.6 Data Analysis

Audio (and/or video) recorded interview data will be transcribed verbatim and imported into the latest version of NVivo software for analysis. A framework analysis approach will be used (Hackett and Strickland 2019). This matrix‐based method provides a transparent and systematic structure that supports the progression from raw data through to explanatory accounts. The process involves five interconnected stages: familiarisation, constructing a thematic framework, indexing and sorting, data summary and display, and mapping and interpretation (Ritchie 2014).

Data analysis will be conducted independently by up to three members of the research team, followed by collaborative discussions to agree on key themes and resolve any differences. This team‐based approach will enhance the rigour and credibility of the findings. Given the culturally situated nature of the study, analysis will be undertaken through a reflexive lens, attending to issues of power, positionality and intersectionality. The diversity of the research team will further enhance the interpretive depth and cultural sensitivity of the analysis.

The study's two theoretical frameworks, The Silences Framework and Intersectionality Theory will underpin the analytical process. The Silences Framework will guide the structuring of the analytical matrix and the identification of these themes that reflect hidden or marginalised narratives, while Intersectionality Theory will shape the interpretation of these themes by situating them within broader structures of power and inequality. This integration ensures that the Framework Analysis is both inclusive and critically attuned to the social contexts of participants.

Findings from this work package will directly inform the design and content of the codesign workshops in Work Package 2, ensuring that participants' insights directly shape the development of actionable, evidence‐informed recommendations.

### 4.5 Work Package 2: Codesign Workshops

This work package will use a modified Delphi approach to develop theory‐informed, pragmatic recommendations aimed at supporting research engagement and capacity among Black nursing academics. Rather than recruiting participants in the traditional sense, the codesign workshops will involve key collaborators who will work in equal partnership with the research team to co‐create actionable, contextually relevant recommendations. These collaborators will contribute both lived experience and institutional insight, fostering a more equitable and grounded process.

Two rounds of codesign workshops will be conducted, adhering to best practice principles to ensure the process remains collaborative, iterative and directly informed by the findings from Work Package 1.

#### 4.5.1 Objectives

The objectives of this work package are to:

1. Explore effective strategies and practices that can be implemented to address these barriers and create supportive research environments.
2. Co‐create theory‐informed, pragmatic recommendations to foster enabling and equitable research environments for Black nursing academics in non‐Russell Group UK universities, with consideration of their transferability to other Black academics and racialised researchers in similar institutional contexts.

#### 4.5.2 Sample Size

Up to 20 key collaborators will be recruited to take part in two rounds of iterative online codesign workshops. Based on prior experience, not everyone who signs up typically attends, so recruiting up to 20 collaborators provides flexibility while maintaining the depth and diversity of perspectives required for meaningful co‐creation.

#### 4.5.3 Eligibility Criteria

Key collaborators will be recruited based on the eligibility criteria outlined in Table 1.

**TABLE 1:** Eligibility criteria for key collaborators.

Category	Eligibility criteria
Demographic	Self‐identify as Black (African, Caribbean or other Black heritage) Institutional leaders and research support staff may participate regardless of ethnicity due to their strategic/operational roles in supporting research
Professional	Academics: Currently in an academic or research role at a UK university, within a health and social care discipline (with or without significant responsibility for research) Institutional Leaders (e.g., Deans, Associate Deans, Heads of Department) and Research Support Staff: Not required to hold academic posts but must have strategic, operational or oversight responsibilities related to research
Geographic & Institutional	Based at a UK university May be affiliated with either Russell Group or non‐Russell Group universities. Representation will be sought from England, Scotland, Wales and Northern Ireland

#### 4.5.4 Key Collaborator Sampling and Recruitment

To ensure a diverse and meaningful recruitment process for the codesign workshops, an adaptive recruitment strategy will be adopted. The research team will engage in strategic outreach in collaboration with the Council of Deans of Health and the Society of Black Academics to identify individuals who can meaningfully contribute to the co‐creation of practical, evidence‐informed recommendations. The Council of Deans of Health will support dissemination through university research offices and nursing departments, while the Society of Black Academics will help engage Black academic audiences within UK higher education institutions.

Key collaborators will be identified using a combination of purposeful, snowballing and convenience sampling techniques. Purposeful sampling will enable the intentional inclusion of individuals with relevant experience and insights aligned with the eligibility criteria. Snowballing will support wider engagement across academic and professional networks, particularly reaching those not easily accessed through initial outreach. Convenience sampling will allow the inclusion of individuals who express early interest and whose availability aligns with the planned workshop dates.

The recruitment process will remain flexible and responsive to ensure a balance of perspectives across professional roles, career stages, institutional contexts and geographical regions. The aim is to engage a diverse and knowledgeable group of collaborators, including Black academics with and without formal research responsibilities, institutional leaders (e.g., Deans, Associate Deans, Heads of Department) and research support staff. All collaborators will be invited to work collaboratively and equitably with the research team throughout the codesign process.

To further extend the reach of the invitation, the research team will also collaborate with professional bodies such as the Royal College of Nursing (RCN) and use platforms like LinkedIn and X (formerly Twitter) to share information widely across academic and professional networks.

Tailored email invitations will be sent on behalf of the University of Staffordshire, inviting eligible individuals to contribute to two rounds of iterative online codesign workshops. Interested individuals will be asked to complete a short electronic expression of interest form, which will also provide the opportunity to raise any queries. Each email invitation will include a project overview and a collaborator information sheet outlining the purpose of the workshops, the nature of their collaborative role and collaborators' rights to withdraw at any stage. All queries will be addressed by the Principal Investigator.

Participation is voluntary. Collaborators will be given a minimum of 24 h to consider their involvement before completing a brief electronic collaboration agreement form for research records. Verbal agreement to participate will also be confirmed and audio recorded at the beginning of each workshop to ensure transparency and mutual understanding of roles.

#### 4.5.5 Codesign Workshop Plan

Two online codesign workshop sessions will be convened with key collaborators who bring lived experience and/or institutional expertise in relation to research capacity among Black nursing academics in the UK.

In the first workshop, anonymised findings from Work Package 1 will be presented to collaborators. This session will provide an opportunity for collaborators to reflect on the findings, explore contextual nuances and begin codesigning preliminary, theory‐informed recommendations.

The second workshop will focus on reviewing and refining these initial recommendations. Collaborators will be asked to evaluate these findings using the APEASE (Acceptability, Practicability, Effectiveness and cost‐effectiveness, Affordability, Safety and Equity) criteria (Presseau et al. 2019). APEASE criteria support intervention designers to make context‐specific decisions about the content and delivery of proposed actions (Atkins 2016). The aim is to produce a set of actionable, contextually relevant recommendations grounded in collaborative consensus.

Each workshop will last a maximum of 3.5 h and will include 15‐min breaks every hour to support wellbeing and active engagement. Sessions will be hosted via Microsoft Teams, a secure online platform, to enhance accessibility and reduce barriers related to geography, workload or caring responsibilities. All workshops will be audio and/or video recorded to ensure an accurate account of the discussions.

To foster meaningful engagement and dialogue, each session will include whole‐group discussions and smaller breakout groups as appropriate. Collaborators will begin in a main session and then move into breakout groups (with not more than five collaborators per group) for focused discussions and task‐based activities.

Each breakout group will be facilitated by a trained member of the research team using a structured facilitation guide. While the guide will support consistency across groups, facilitators will also allow space for emergent insights and diverse perspectives. After each breakout, the full group will reconvene to share reflections and synthesise key points, ensuring that all voices are acknowledged and valued. Throughout the workshops, the research team will actively address potential power dynamics, promoting a collaborative and respectful atmosphere.

#### 4.5.6 Codesign Workshop Process and Collaborative Synthesis

The workshops in this work package serve as both a mechanism for engagement and as a site for collaborative synthesis. Rather than applying traditional post hoc data analysis techniques, this stage of the study adopts a participatory approach in which interpretation, sense‐making and recommendation development are embedded within the codesign process itself, informed throughout by the study's theoretical framework.

In both workshops, collaborators will engage in structured discussions within facilitated breakout rooms. These sessions will support critical reflection on findings from Work Package 1 and help to refine and prioritise the emerging recommendations. The breakout rooms will encourage and promote contextual understanding, generation of diverse ideas and equitable contributions from all collaborators.

Outputs from each breakout room will be shared during a plenary session, where the wider group will collectively begin to shape initial recommendations in Workshop 1 and refine them in Workshop 2. These discussions will be documented in real time using shared visual tools (e.g., whiteboards or collaborative notes) and supported by researcher field notes and workshop recordings.

Given the participatory nature of the workshops, particular attention will be paid to ensuring that the synthesis process remains grounded in the voices and lived experiences of collaborators. Here, the Silences Framework informs the creation of an environment that encourages seldom‐heard perspectives, while Intersectionality Theory ensures that intersecting identities are considered in facilitation and interpretation. Together, these frameworks guide the collaborative synthesis and prioritisation of recommendations through the modified Delphi process, ensuring outputs are contextually grounded and sensitive to equity and inclusion. Where appropriate, member checking or follow‐up feedback loops may be used to validate key insights and enhance the trustworthiness of the outputs.

Following the workshops, the research team will synthesise the outputs into a coherent narrative, capturing the development and evolution of the recommendations and the collaborative decision‐making process. This synthesis will form the basis of the final output of Work Package 2: a set of actionable, theory‐informed and consensus‐based recommendations to enhance research capacity for Black nursing academics.

#### 4.5.7 Codesign Outputs: Recommendations and Position Statement

The codesign workshops will culminate in a set of actionable, theory‐informed recommendations to enhance research capacity for Black and other racialised researchers in the UK. These recommendations will reflect the diverse perspectives and lived experiences of the collaborators, including Black academic staff from nursing and other health and social care disciplines, institutional leaders and research support staff.

Co‐created through structured dialogue and informed by the findings from Work Package 1, the recommendations will focus on addressing systemic barriers and opportunities within non‐Russell Group universities. The APEASE criteria will guide discussion to ensure outputs are contextually relevant, grounded in empirical evidence and feasible for implementation across UK higher education institutions. This consensus‐based approach will ensure the final recommendations reflect a collective commitment to advancing equity in research.

A Position Statement will also be developed to accompany the recommendations. This statement will articulate a shared vision for advancing racial equity in research leadership and capacity building. It will highlight the urgency of addressing persistent inequalities and serve as a call to action for institutions and policymakers to engage with and implement the recommendations in a meaningful and sustainable way.

Together, these outputs will be synthesised into a coherent, actionable framework that can guide institutional practices, inform policy development and drive systemic change in the support and progression of Black and other underrepresented academics.

### 4.6 Data Management

Audio (and/or video) recorded interviews will be transcribed verbatim, and recordings will be destroyed once transcripts have been verified and corrected. It is expected that Microsoft Teams will transcribe video files but if this is not done sufficiently, the recordings will be sent to a third‐party company. This company will be a preferred supplier of University of Staffordshire, with whom all necessary due diligence will have taken place in regard to data protection. This will be clearly stated in the participant information sheet. All electronic data will be securely stored on a password‐protected, encrypted laptop owned by University of Staffordshire. Personally identifiable data, such as consent and codesign collaboration agreement forms, will be collected digitally and stored within Microsoft Teams (on a SharePoint server owned and operated by University of Staffordshire). These will be stored separately from anonymised research data. Interview participants and codesign key collaborators who request a copy of the study findings will need to share their email addresses which then become personally identifiable data. These will be kept up to when the study findings are written and sent out to such participants/collaborators.

As an output for the codesign workshops, collaborators' names, roles and affiliations will be included in the final Position Statement, as part of the study's dissemination. Collaborators who agree to be named in the final Position Statement will have provided their permission whilst completing the collaborator agreement form. This data will be stored securely and used only for authorship and public recognition within the Position Statement. If collaborators choose not to be named, their contributions will still be included anonymously.

Data management will comply with UK GDPR 2018 and the Data Protection Act (2018), and University of Staffordshire data storage policy. Anonymised research data may be retained indefinitely, following open access expectations, but for at least 10 years after study completion. Only the study team will have access to this data.

### 4.7 Ethics Considerations

Both potential interview participants and key collaborators will be approached in a manner that respects their privacy and data protection rights.

For Work Package 1, potential participants will receive an information sheet and be given sufficient time to consider their participation before completing an electronic consent form for research records. Verbal consent will also be recorded prior to the interview. Participation will be entirely voluntary, and individuals may withdraw up to 7 days after the interviews after which their data will be anonymised and data analysis will have commenced.

All participant data will be treated confidentially and securely. Personally identifiable information will not be shared outside the immediate research team unless required by safeguarding obligations. Personal data will be stored separately from coded research data, and only members of the research team will be able to link the two.

For Work Package 2, additional ethical considerations apply given the participatory and collaborative nature of the work. Collaborators will receive an information sheet and be required to complete an agreement form. Due to the interactive nature of the workshops, individual contributions cannot be isolated or withdrawn after involvement. This limitation will be clearly communicated in advance and reiterated at the start of each workshop.

To support inclusive and safe contributions, ground rules for confidentiality, respectful dialogue and psychological safety will be co‐established at the beginning of each workshop. While efforts will be made to anonymise contributions in written notes and analysis where appropriate, collaborators will be informed that full anonymity cannot be guaranteed because of the group dynamics.

Importantly, collaborators will need to agree to the inclusion of their names, roles and institutional affiliations in the final Position Statement. This will be presented as an opportunity to recognise their leadership contributions, with each collaborator given the option to decline public attribution. No personally identifiable information will be published without express permission from collaborators.

### 4.8 Methodological Rigour, Reflexivity and Positionality

Ensuring rigour and reflexivity is central to the credibility and trustworthiness of the proposed study. This study adopts a theory‐informed and team‐based approach to design, data collection and analysis, supported by tools such as a scripted interview schedule, structured facilitation guides and framework analysis using NVivo software. These strategies promote consistency across all stages of the research while safeguarding the integrity and cultural relevance of the data.

Reflexivity has been integrated from the earliest stages. The study was developed in collaboration with a Protocol Development Advisory Group comprising Black nursing academics, whose insights helped shape a culturally sensitive, inclusive and ethically grounded research design. The research team will engage in collective reflexive practice, acknowledging how positionality including race, gender and institutional role may influence interpretation and researcher‐participant dynamics.

A key strength of the study is the racial and professional diversity of the research team. The interviews will be conducted by Black nursing academics, which enhances cultural competence and supports rapport with participants. In instances where this is not possible, detailed interview scripts and reflexive briefings will help maintain consistency and ensure respectful engagement.

The project is underpinned by Intersectionality Theory and The Silences Framework, both of which guide the study's epistemological stance and interpretive lens. These frameworks make visible the often‐overlooked intersections of race, gender and professional marginalisation, and reinforce the commitment to ensuring participant voices remain central throughout the research process.

Through these methodological choices, the study aims to uphold both scientific rigour and ethical accountability, ensuring that the findings are both trustworthy and transformative.

## 5 Discussion

This study is designed to address the systemic barriers that limit Black nursing academics' engagement with research within non‐Russell Group UK universities. Despite the increasing diversity of the nursing workforce, Black academics remain significantly underrepresented in research leadership, funding success rates and scholarly output (Higher Education Statistics Agency 2024; UK Research and Innovation 2020). Such underrepresentation not only restricts the scope of nursing research but also hinders career progression, contributing to a healthcare education system that does not fully reflect the needs of an increasingly multicultural UK population (Banister et al. 2020; Whitfield‐Harris and Lockhart 2016).

By exploring the specific barriers to research engagement faced by Black nursing academics, this study is expected to generate evidence that can inform structural change. The two‐work package design, integrating qualitative interviews and structured codesign workshops ensures that the lived experiences of Black academics directly shape the study's outputs. This design is anticipated to enhance both the relevance and feasibility of the recommendations generated, while embedding collaboration and reflexivity at every stage.

The potential implications of the study extend beyond institutional reform. It is anticipated that findings will provide insights that contribute to the development of culturally competent care models and support the decolonisation of nursing curricula (Crooks et al. 2021; Gimbel et al. 2017), challenging Eurocentric frameworks and broadening the evidence base (Whitfield‐Harris and Lockhart 2016; Iheduru‐Anderson et al. 2022). Strengthening the presence of Black academics in research leadership is expected to help foster a more diverse academic workforce and inform the creation of a more equitable and responsive health and social care system (Ede et al. 2024).

## 6 Recommendations

The study is designed to generate actionable, theory‐informed recommendations to enhance research capacity for Black nursing academics in non‐Russell Group universities. These recommendations will be codesigned with key collaborators to ensure they are grounded in lived experiences shaped by practical expertise, thereby enhancing both relevance and feasibility.

It is anticipated that recommendations will address systemic barriers across several areas. First, they may propose approaches to ensuring equitable access to research funding, including tailored institutional support mechanisms and transparent allocation processes. Second, they are expected to emphasise the importance of mentorship and leadership pathways that are sensitive to the challenges faced by Black nursing academics, recognising the significance of role models and networks in supporting career progression. Third, they are likely to encourage the development of supportive institutional research cultures, including workload adjustments, recognition of research activity and equity‐focused strategic planning.

Finally, recommendations are expected to promote culturally competent research practices within academic and policy frameworks, such as inclusive curriculum design, equitable authorship practices and diverse collaborations. Collectively, these outputs aim to advance an inclusive and representative research environment in UK higher education.

## 7 Conclusion

This proposed study represents a timely and necessary intervention to address the persistent underrepresentation of Black nursing academics in research. By adopting culturally sensitive methodologies and centring lived experiences, the study is designed to produce actionable, theory‐informed recommendations that foster equitable research environments within UK higher education.

The anticipated implications extend across practice, policy and research. For practice, the study is expected to generate strategies to embed culturally competent approaches in nursing education and research. For policy, it is designed to provide an evidence base to support action on inequities in research funding, leadership opportunities and institutional support. For research, the study will offer a framework for examining the experiences of other underrepresented groups, both in the UK and internationally, and for assessing the impact of equity‐focused interventions over time.

By situating the UK experience within global debates on equity in nursing and higher education, the study is designed to contribute to the international evidence base. In doing so, it is anticipated that the study will help lay the groundwork for sustainable institutional change and promote the development of an inclusive and responsive academic and healthcare research landscape worldwide.

## Author Contributions

All listed authors made substantial contributions to the study design and development of this protocol, in accordance with the ICMJE criteria for authorship. Y.A. and S.J. conceptualised the study. All authors contributed to drafting the manuscript, critically revised the content for intellectual accuracy and clarity and approved the final version for submission.

## Ethics Statement

Ethics approval for this study has been granted by the University of Staffordshire Research Ethics Committee (Reference: SU_24_125).

## Conflicts of Interest

The authors declare no conflicts of interest.

## Data Availability

The authors have nothing to report.
